# Do Genetic Drift and Gene Flow Affect the Geographic Distribution of Female Plants in Gynodioecious *Lobelia siphilitica*?

**DOI:** 10.3390/plants11060825

**Published:** 2022-03-20

**Authors:** Hannah J. Appiah-Madson, Eric B. Knox, Christina M. Caruso, Andrea L. Case

**Affiliations:** 1 Department of Marine and Environmental Science, Ocean Genome Legacy Center, Northeastern University, Nahant, MA 01908, USA; 2 Department of Biology, Indiana University, Bloomington, IN 47405, USA; eknox@indiana.edu; 3 Department of Integrative Biology, University of Guelph, Guelph, ON N1G 2W1, Canada; carusoc@uoguelph.ca; 4 Department of Biological Sciences, Kent State University, Kent, OH 44240, USA; acase@kent.edu

**Keywords:** gene flow, genetic drift, geographic structure, gynodioecy, *Lobelia siphilitica*, non-selective mechanisms, sex ratio

## Abstract

Variation in population sex ratio is particularly pronounced in gynodioecious angiosperms. Extremely high female frequencies in gynodioecious populations cannot be readily explained by selective forces alone. To assess the contributions of drift and gene flow to extreme sex-ratio variation, we documented sex ratio and population size in 92 populations of *Lobelia siphilitica* across its range and genotyped plants using plastid and nuclear genetic markers. Similarity in spatial patterns of genetic and demographic variables may suggest that drift and/or gene flow have contributed to population sex-ratio variation in *L. siphilitica*. We found strong spatial structuring of extremely high female frequencies: populations with >50% female plants are restricted to the south–central portion of the range. However, we did not detect any spatial structuring in population size nor metrics of genetic diversity, suggesting that extreme variation in female frequency is not strongly affected by drift or gene flow. Extreme sex-ratio variation is frequently observed in gynodioecious plants, but its causes are difficult to identify. Further investigation into mechanisms that create or maintain the spatial structure of sex ratios in gynodioecious species will provide much needed insight.

## 1. Introduction

Understanding the maintenance and partitioning of within-species phenotypic diversity is a critical goal of evolutionary ecology. One common type of within-species phenotypic diversity is variation in population sex ratio [1,2,3,4,5]. Among genetically based sexual systems in angiosperms, gynodioecy—the co-occurrence of female and hermaphroditic plants within populations—is a particularly good system to evaluate. This is because sex-ratio variation is particularly pronounced in gynodioecious species, ranging from 0 to 100% female in some taxa. Thus, insights into this system are key to understanding the evolution of sexual systems in flowering plants and the mechanisms that create and maintain variation in population sex ratio [6].

Sex ratios in natural populations of gynodioecious angiosperms can be strongly female biased (>50% female), but populations are more often hermaphrodite biased (averaging ~25% female across species) [5]. This pattern of extremely variable but low average female frequencies is especially common in gynodioecious species with complex cytonuclear sex determination [7]. In these species, female plants carry male-sterility genes in their mitochondrial genomes (i.e., cytoplasmic male sterility, or CMS) [8,9], whereas hermaphrodites either do not carry CMS or carry CMS plus one or more nuclear male-fertility restorer (*Rf*) alleles, allowing them to produce pollen despite carrying CMS [10,11,12,13]. Cytoplasmic male-sterility genes and *Rf* alleles enter populations through either mutation or gene flow. However, it is the maintenance of polymorphism at CMS and/or *Rf* loci within populations that results in the production of female plants. This is because random mating is likely to result in mismatched CMS and *Rf* alleles in offspring, especially when there are many unique alleles at CMS and/or *Rf* loci [3]. Polymorphism at CMS and *Rf* loci appears to be primarily maintained long-term by balancing selection (specifically, negative frequency-dependent selection) [14,15,16] under a broad range of demographic conditions. However, modeling [16] and recent empirical work [14] show that balancing selection alone cannot readily account for extremely high female frequencies.

Non-selective forces, including genetic drift and gene flow, could contribute to extreme sex-ratio variation in gynodioecious species through effects on CMS–*Rf* polymorphism [3,17]. The frequent origin of novel CMS genes via mitochondrial mutation is expected to result in locally high female frequencies [14]. However, this depends on novel CMS types becoming established and spreading readily within populations [18]. Genetic drift can affect the level of polymorphism in a population through the random loss or fixation of particular CMS genes or *Rf* alleles, especially in smaller populations [19,20,21,22]. This means that small populations may have a wider range of female frequencies, and thus a higher average, compared to larger populations. In contrast to drift, gene flow is expected to maintain female plants regionally by (re-)introducing CMS–*Rf* polymorphism. This is especially the case when proximal populations harbor unique assemblages of CMS genes and *Rf* alleles [23,24,25]. Importantly, gene flow and genetic drift may vary in their effects across a species range. Specifically, these forces are expected to contribute to central–peripheral structuring of genetic diversity [26,27,28,29]. The ‘abundant-center’ model predicts that central populations are larger, older, less susceptible to genetic drift, and better connected via gene flow compared to populations in peripheral locations, all of which can increase overall genetic diversity in central populations when compared to those at the periphery [29]. If the higher overall genetic diversity predicted for central populations includes higher polymorphism at sex-determining loci, we would expect higher female frequencies in central relative to peripheral populations. 

**Figure 1:**
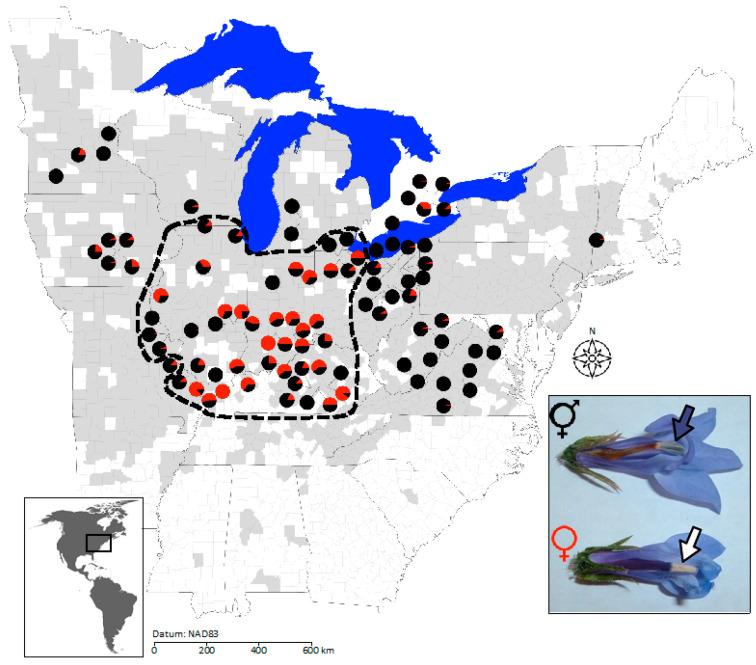
Distribution and population sex ratios of 92 eastern North American *Lobelia siphilitica* populations sampled in 2009 and 2011. Pie charts reflect the frequency of female (red) to hermaphroditic (black) plants for each population, or average frequencies if the population was sampled in both years. Populations inside the dashed black line were considered central based on level II ecoregions [32]. Shaded US counties show the known distribution of *L. siphilitica* based on online specimen collection databases (see text for details). Female and hermaphrodite flowers of *L. siphilitica* are easily distinguishable (photo inset; photo credit Maia F. Bailey, used with permission).

To examine the causes of extreme population sex-ratio variation, we studied the range-wide distribution of female plants in gynodioecious *Lobelia siphilitica* (Campanulaceae). *Lobelia siphilitica* L. var. *siphilitica* is a perennial wildflower that occurs throughout northeastern North America (Figure 1, grey shading) [30]. Our previous research documented several aspects of *L. siphilitica* sex-ratio variation that make it an excellent study system. Two previous censuses of *L. siphilitica* populations documented sex ratios ranging from 0 to 100% female, with higher female frequencies at lower latitudes [14,31]. However, Caruso and Case [31] included few populations in the southern half of the species range, making it insufficient to fully document spatial structuring of female frequencies, particularly central–peripheral patterning. Adhikari et al. [14] examined the effects of balancing selection on population sex ratio in *L. siphilitica* and found that this process can explain sex ratios up to but not above ~50% female. Populations with extremely high female frequencies contained rare mitochondrial haplotypes, suggesting elevated rates of CMS origin and turnover and the absence of balancing selection. Consequently, gene flow and genetic drift may maintain rare haplotypes and novel CMS genes in some sites, but not others. 

In this study, we sampled and genotyped plants from 92 populations across a central–peripheral gradient to capture the demographic (i.e., population size), geographic, and genetic correlates of female frequency. To test the hypothesis that genetic drift and/or gene flow have contributed to demographic and geographic structuring of high female frequencies in *L. siphilitica*, we used plastid (pt) and neutral nuclear genetic markers. We note that, although strong linkage disequilibrium (LD) between pt and mitochondrial (mt) variation is expected, it has been shown to be relatively weak in *L. siphilitica* [14], meaning that variation in the pt genome is more ‘neutral’ with respect to sex-determining loci than under high pt–mt LD. We tested for statistical associations between population sex ratio, population size (a proxy for genetic drift), and neutral genetic diversity, as well as estimated rates of gene flow. Strong statistical associations among demographic and genetic metrics would suggest that genetic drift and/or gene flow have contributed to the present-day spatial structuring of population sex ratios in *L. siphilitica*, especially if multiple metrics show similar patterns of geographic structuring.

## 2. Results

### 2.1. Data Characteristics

#### 2.1.1. Demography

Both population size and female frequency were highly variable. Population size ranged from two to several thousand flowering stems per site (mean = 182; *n* = 91 populations). Female frequency varied between 0 and 1 (mean = 0.218; *n* = 92 populations; Figure 1; Table S1). Upon binning by sex ratio for categorical analysis, our sample included relatively even numbers of populations with 0% (*n* = 26 populations), 0.1–10% (*n* = 23 populations), 10.1–50% (*n* = 23 populations), and >50% female plants (*n* = 20 populations). Two-thirds of the sampled populations were ‘small’ (<100 plants; *n* = 61 out of 92 populations).

#### 2.1.2. Plastid Sequence Data

**Figure 2:**
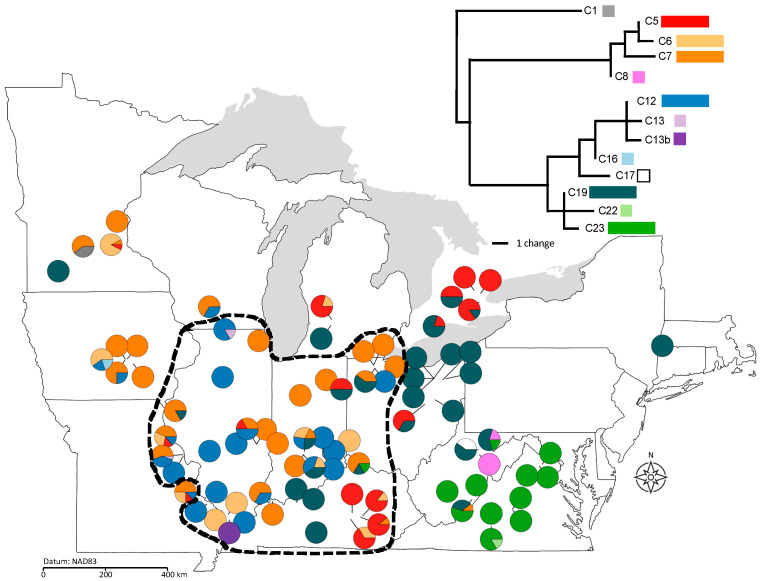
Distribution of 13 plastid haplogroups within and among populations of *Lobelia siphilitica* from 2009 and 2011. Colors correspond to haplogroups shown in the inset. Between 2 and 46 individuals (mean = 7.01) were sampled in each population. The black dashed line indicates delineation between central and peripheral populations based on ecoregion. Inset shows the phylogram for the 13 pt haplogroups (see: Figure S2). Long bars indicate common haplogroups and short bars indicate rare haplogroups.

We sequenced one hypervariable intergenic region of the plastid genome (*psb*K–*rps*16; Figure S1). Out of 142 complete sequences of the *psb*K–*rps*16 region, we identified 120 unique pt haplotypes. Individual populations contained 1–13 unique haplotypes based on the full pt marker sequence. Excluding variation within a hypervariable minisatellite locus (see justification in Methods), these 120 haplotypes were condensed into 13 haplogroups using parsimony (Figure S2). The analysis of pt variation presented here is based on these 13 inferred haplogroups. Six common haplogroups accounted for 95% of sequenced individuals (Figure 2). The remaining seven haplogroups were restricted, each occurring in fewer than four individuals in our sample. Six of these seven haplogroups occurred in only one or two populations each. In two-thirds (67%) of our sampled populations, we found only one haplogroup represented and the maximum number in a single population was 4 (mean = 1.106; Figure 2, Table S1). Plastid haplogroup diversity (*h*) ranged from 0 to 0.81 (mean = 0.187) across populations.

#### 2.1.3. Nuclear Microsatellite Diversity

We detected 12, 17, and 21 alleles for our three nuclear microsatellite loci (*Lob6*, *Lob9*, and *Lobtri1*), respectively, for a total of 50 unique alleles (Table S2) and 4.2–5.8 alleles per population (*N*_a_ in Table S3). All but three populations were polymorphic at all three loci and observed (*H*_o_) and expected (*H*_e_) heterozygosities were moderate (Table S3). Across populations, pt haplogroup diversity (*h*) was significantly correlated with nuclear allelic diversity (*h* vs. *N*_a_: linear regression, *r* = 0.289, df = 80, *p* = 0.0085; *h* vs. *N*_e_: linear regression, *r* = 0.225, df = 80, *p* = 0.042) but not with heterozygosity (*h* vs. *H*_o_: linear regression, *r* = 0.04, df = 80, *p* = 0.10). 

### 2.2. Geographic Structure in Population Sex Ratios, Population Size, and Neutral Genetic Diversity

Based on Moran’s I, female frequency was highly spatially structured (Moran’s I = 0.289, *p* < 0.00001; Figure 1). Female plants were more abundant at lower latitudes (linear regression between sex ratio and latitude, *r*^2^ = 0.096, df = 90, *p* = 0.0027) and mid-longitudes (non-linear regression between longitude and sex ratio, *r*^2^ = 0.20, df = 90, *p* < 0.0001). Consequently, mean female frequency was more than 7-fold higher in central populations (38.4% female, *n* = 46) than peripheral populations (5.3% female, *n* = 46; 2-tailed *t*-test, *t* = −7.1, df = 90, *p* < 0.0001), and populations with >50% female plants (*n* = 20) were exclusively central. 

In contrast to sex ratio, population size was not spatially structured (Moran’s I = 0.05, *p* = 0.23), and varied little in size across latitudes (linear regression, *r^2^* = 0.098, df = 89, *p* = 0.0025). Notably, while both population size and sex ratio varied with latitude, population size did not account for any variation in population sex ratio (linear regression, *r^2^* = 0.006, df = 89, *p* = 0.45), and there was no significant difference in mean population size between central and peripheral populations (2-tailed *t*-test, *t* = 0.07, df = 89, *p* = 0.95).

We detected no spatial structure in pt haplogroup diversity (*h*; Moran’s I = −0.055, *p* = 0.44), nor in microsatellite diversity (*H_o_*, *N_a_*, or *N_e_*; Moran’s I = 0.004–0.08, *p* > 0.09). Plastid haplogroup diversity (*h*) did not vary with sex ratio either continuously (linear regression, *r^2^* = 0.0022, df = 81, *p* = 0.67) or categorically (ANOVA, *F*_3,79_ = 0.73, *p* > 0.53). Likewise, *h* did not differ significantly between central and peripheral populations (1-tailed *t*-test, *t* = 0.24, df = 81, *p* = 0.59), nor with population size (linear regression, *r^2^* = 0.00003, df = 81, *p* = 0.96). For nuclear microsatellites, *N*_a_ and *N*_e_ were slightly higher in large vs. small populations (1-tailed *t*-tests, *t* = 2.10–3.89, df = 88, *p* < 0.019), but other groupings (including population sex ratio) showed no significant variation in any metric of nuclear genetic diversity.

### 2.3. Population Differentiation and Gene Flow

Our results are consistent with a high degree of population genetic differentiation but low rates of contemporary gene flow. Populations were highly differentiated (pt *F*_ST_ = 0.75) and relatively isolated (estimated *N*_e_*m* = 0.166) based on pt haplogroups, consistent with effects of historical migration on population structure. Likewise, Mantel correlations revealed slight but significant isolation by distance (*r* = 0.112, *p* = 0.01) and notably high pairwise genetic distances for even the most proximal population pairs (Figure S3). Average pairwise *F*_ST_ for nuclear microsatellites, which should reflect more recent gene flow, indicates moderately strong population differentiation (nuclear *F*_ST_ = 0.222) and low rates of migration (*N*_e_*m* = 0.884; Table S3). Isolation by distance was comparable to that shown for the pt data, suggesting that gene flow is not frequent (Mantel correlation: *r* = 0.246, *p* = 0.01; Figure S3).

### 2.4. Population Genetic Structure in Relation to Variation in Sex Ratios, Population Size, and Geography

Five of the six common pt haplogroups were significantly clustered based on both their presence in populations (Moran’s I values = 0.163–0.41, *p* < 0.00024) and their relative abundance in populations (Moran’s I values = 0.22–0.468, *p* < 0.0001). These five spatially clustered haplogroups were either predominantly eastern or predominantly western (relative to the midpoint of the sampled range at −85.45 longitude; 2-tailed *t*-tests comparing the mean longitude of populations with and without each haplogroup, *t* = −5.26–4.47, df = 81, all *p* < 0.0245). One of these five structured haplogroups (C23) showed additional north–south structuring, with all but one case restricted to the southeastern corner of the sampled range in West Virginia and Virginia (Figure 2; 2-tailed *t*-test comparing the latitude of populations with and without C23, *t* = −3.49, df = 81, *p* < 0.0008). However, for five of the six common haplogroups, the presence vs. absence of each haplogroup was not associated with differences in sex ratio between populations, suggesting no relationship between specific pt haplogroups and female frequency (2-tailed *t*-tests, *t* = −0.84–1.83, df = 81, *p* > 0.07). Although populations that contained C23 had significantly lower female frequencies than those without C23 (3.9% vs. 26.7%; 2-tailed *t*-test, *t* = −5.58, df = 81, *p* = 0.0001), these sites are likely low female because they are almost exclusively peripheral, not because they harbor haplogroup C23. 

Population genetic structure is poorly explained by demography and geography. Little molecular variation was explained by any among-group comparisons in the AMOVAs. However, the few small but significant effects were geographic rather than demographic (Table 1). Central vs. peripheral groupings explained ~10% of pt molecular variance; while peripheral populations contained a greater number of unique haplogroups, the haplogroups present in central populations were more divergent from each other in sequence (Figure 2). An exceedingly small but significant portion of nuclear diversity was explained by central vs. peripheral location (~2%) but these patterns were not borne out by any post hoc comparisons among groups. Grouping populations by sex ratio or population size in the AMOVAs did not account for any pt molecular variation, and sex ratio alone accounted for a statistically but not biologically significant portion of nuclear diversity (~1%), reflecting populations without female plants having slightly fewer alleles than populations with female plants (ANOVA, *F*_3,85_ = 3.04–4.77, *p* < 0.033).

## 3. Discussion

Our goal was to determine the contribution(s) of genetic drift and gene flow to extremely high female frequencies in populations of gynodioecious *L. siphilitica*. Sex ratios were strongly spatially autocorrelated, with populations over >50% female restricted to the south–central portion of the sampled range (Figure 1). However, variation in female frequency was not significantly associated with variation in population size nor with metrics of pt or nuclear genetic diversity (Table 1 and Table 2). Thus, smaller populations did not contain a higher proportion of female plants than larger populations, contrary to results reported in Caruso and Case [31]. Additionally, while individual pt haplogroups were spatially structured, we found no association between pt haplogroup structure and variation in population female frequency. Overall, contrary to our hypothesis, we found that neither genetic drift nor gene flow have likely contributed to the extreme sex-ratio variation within this species.

While we observed strong central–peripheral structuring of female frequencies, none of the other predictions of the abundant-center hypotheses were supported by our data. We did not observe higher levels of pt or nuclear genetic diversity in central populations nor in populations with more female plants. Central populations were not larger in size and did not experience more gene flow than peripheral populations. Combined with results of our previous work [14], these results suggest that high female frequencies within populations of *L. siphilitica* are most strongly associated with unique mitochondrial haplotypes rather than high cytonuclear polymorphism overall, as is commonly predicted [10,23,33]. While we are able to associate unique mitochondrial diversity with extreme female frequencies, further research is needed to understand why females are concentrated in the center of the species range.

Our finding that genetic drift and gene flow have not contributed significantly to extreme sex-ratio variation in *L. siphilitica* is consistent with studies of some gynodioecious species but not others. They are consistent with results from *Beta vulgaris* ssp. *maritima* (Chenopodiaceae), where non-selective forces also do not strongly affect population sex ratios, despite high population differentiation and low migration [16,34]. However, our results are not consistent with studies of several other gynodioecious species, which have found evidence that genetic drift [19,22] or gene flow [25] can contribute to variation in female frequencies. Consequently, there is currently little consensus on the role of drift and gene flow in structuring sex ratios of gynodioecious angiosperms. Further investigations may point to other features of particular gynodioecious species that make their sex ratios more or less susceptible to these non-selective forces. However, comprehensive explanations for population sex-ratio variation would need to explain cases where sex ratios are not only variable but also spatially structured, which has been documented in *L. siphilitica* as well as several other gynodioecious species [22,25,35,36]. 

We documented strong central–peripheral structuring of population sex ratios in *L. siphilitica*: female plants were much more frequent in southern-central populations compared to northern or peripheral populations (Figure 1). A range-wide survey of gynodioecious *Nemophila menziesii* (Boraginaceae) also found that female plants were more common in the central portion of the species range [25]. It is yet unclear whether central–peripheral structuring of sex ratios is common among gynodioecious plants because few species have been censused and the spatial distribution of sampled populations has been typically restricted relative to the species’ geographic range. Three studies of other gynodioecious species report latitudinal gradients, where female plants either increase (*Plantago maritima* (Plantaginaceae) [22]; *Kallstroemia grandiflora* (Zygophyllaceae) [35]) or decrease with latitude (*Lobelia spicata* (Campanulaceae) [36]), and a fourth found no spatial structure in population sex ratios (*Beta vulgaris* ssp. *maritima* [37]). However, we note that in each of these studies, the sampled population sites were either mostly ‘peripheral’ [22,35,36] or mostly ‘central’ [37], such that the observed latitudinal gradients may potentially be consistent with central–peripheral structuring. If so, all but one of these species would show higher female frequencies in the range center. Including both central and peripheral populations in future studies would help establish whether central–peripheral structuring of sex ratios is common in gynodioecious species. 

While genetic drift and gene flow can reflect contemporary population dynamics, present-day populations also bear the signatures of historical processes, particularly in northern-temperate species such as *L. siphilitica* that occupy previously glaciated areas. The significant spatial autocorrelation we observed for individual pt haplogroups (Figure 2) suggests a legacy of structured post-glacial migration/dispersal that does not correspond to the observed south–central structuring of extreme female frequencies (Figure 1). Post-glacial recolonization has been shown to structure genetic and phenotypic diversity in many plant species throughout the northern hemisphere [38], including confamilial *Campanulastrum americanum* (Campanulaceae) [39,40]. The significant geographic structure of individual pt haplogroups that we observed here is potentially consistent with the legacy of post-glacial migration/dispersal, although there was no clear correspondence of pt haplogroup structure and population sex ratios. This lack of correspondence in spatial structure suggests that, like contemporary drift and gene flow, historical migration/dispersal has not strongly affected the present-day distribution of female plants. Compared to *C. americanum*, the pt spatial genetic structure in *L. siphilitica* is less pronounced across the species range [39]. *Lobelia siphilitica* also exhibits high within-population pt diversity relative to *C. americanum*; several of the pt haplogroups were present range wide and even proximal *L. siphilitica* populations were highly genetically differentiated. A more fine-grained assessment of historical biogeography in this species would help evaluate whether historical events may have contributed to the geography of population sex ratios.

We note that plants in only one of our populations—BT in northern Minnesota—carried pt genomes from haplogroup C1. Unpublished work by E.B. Knox has shown that this particular haplogroup is present in *L. siphilitica* var. *ludoviciana* in parts of the range where *L. siphilitica* var. *siphilitica* is absent (see: Supplemental Information). Collections records from this area of Minnesota document the presence of both varieties. Thus, it is possible that the BT population contains separate plants from both varieties, and we happened to sample some *L. siphilitica* var. *ludoviciana*. Alternatively, there may be some degree of introgression of *L. siphilitica* var. *ludoviciana* plastids from C1 into *L. siphilitica* var. *siphilitica*. We cannot distinguish between these two scenarios using our data, but both present interesting explanations for the observation of the highly distinct haplogroup C1 in our sample.

While our results show that population sex ratios in gynodioecious *L. siphilitica* are geographically clustered, we found little evidence that non-selective forces have significantly contributed to this clustering. Thus, the concentration of high-female populations in the south–central portion of the species range remains unexplained. Previous studies in *L. siphilitica* point to two mechanisms associated with high female frequencies, both of which are associated with variation in sex-determining genes and may operate in spatially variable ways. The first mechanism invokes pleiotropic fitness costs of male-fertility restorers (*Rf*), which are inferred to be greater in *L. siphilitica* populations that host more female plants [41,42,43]. While theoretical models predict that higher pleiotropic costs of *Rf* should result in more female plants [33], there is yet little understanding of what causes variation among populations in the magnitude of cost. If the causes of variation in cost are affected by environmental conditions [15], then context-dependent variation in cost may account for geographically structured variation sex ratios. The second mechanism involves the birth rates of the mitochondrial male-sterility (CMS) genes that cause plants to be female. *Lobelia siphilitica* populations with more female plants host more rare mitochondrial haplotypes likely to carry novel sterility genes [14]. If mechanisms maintaining male-sterility genes are affected by environmental conditions, then this context-dependent variation may account for spatial structure in population sex ratios. 

In conclusion, we see strong spatial structuring of extremely high female frequencies in *L. siphilitica* that is not readily explained by the effects of genetic drift or gene flow. Given that female plants are largely geographically restricted to the southern-central portion of the species range, environmental conditions may play a key role in sex-ratio patterns. Other gynodioecious species studied to date differ from each other in the role of selective and non-selective processes affecting sex-ratio variation, the range of female frequencies observed among populations, and the degree of spatial/geographic structuring of population sex ratios [5]. This lack of consensus has yet to be explained. Further investigation into mechanisms that create or maintain the spatial structure of extreme female frequencies in *L. siphilitica* (as well as other gynodioecious species) will provide much needed insight into how evolutionary forces cause populations to become differentiated.

## 4. Materials and Methods

### 4.1. Study System

*Lobelia siphilitica* L. var. *siphilitica* (Campanulaceae) is a perennial wildflower that occurs throughout northeastern North America (Figure 1, grey shading) [30]. *Lobelia siphilitica* var. *ludoviciana* A. DC. [44] occurs farther to the west; and for simplicity, we refer to the autonymic eastern variety just as *L. siphilitica*. Sex is genetically determined and cytonuclear: male-sterility genes in the mitochondrial genome render plants female unless plants carry nuclear loci that suppress the expression of male sterility [11]. The sex morphs are easily identified in the field: hermaphrodite anther cylinders are dark gray-purple, while female anther cylinders are papery white and lack pollen (Figure 1, inset). Individual plants rarely produce flowers of both sexes (i.e., a gynomonoecious phenotype). While *L. siphilitica* is self-compatible, temporal separation between pollen dispersal and maturation of the stigma limits selfing in hermaphrodites to geitonogamy (selfing between flowers on the same plant) [11]. In natural populations, plants flower between July and October and both sexes produce dozens of flowers [45]. Seeds are dispersed passively from dry, dehiscent capsules approximately a month after flowering with each plant producing several thousand seeds [45].

### 4.2. Range-Wide Sampling of Population Size and Sex Ratio

Using specimen-collection databases (sources listed in Supplemental Information), we located 92 populations of *L. siphilitica* across its range. We obtained samples from 71 of these sites between July and September of 2009 and from 21 new sites plus 14 resampled sites in 2011. Very few sites were ambiguous in terms of delimiting clusters of *L. siphilitica* plants as populations; in this analysis, we considered plants to be in separate populations when one patch was not visible from another. Along with GPS coordinates at each site, we estimated the adult population size by counting flowering stems; we did not include immature, non-flowering leaf-rosettes in estimates of population size. We determined population sex ratio using floral morphology. In 18 populations, a few individual plants produced both floral sex phenotypes (36 out of 13,172 plants that were sexed); these ‘partially sterile’ (gynomonoecious) individuals were included in our analyses but were not considered to be ‘female’ in the calculation of female frequency.

We definitively determined total number of plants and sex ratios for 82 of the 92 sites (2–696 individuals per site). For six of seven additional sites hosting very large populations and three additional populations extending into private property, we estimated population size by counting a subset of flowering stems and estimating the total population size by eye based on area and plant density. Female frequency for all seven very large and three partially inaccessible populations was determined based on a subsample of at least 100 plants along transects or on all accessible individuals, respectively. For the 14 populations resampled in 2011, analyses were conducted with the 2009, 2011, and averaged population sizes and sex ratios; sampling year did not affect the results, so we present data based on averaged population sizes and sex ratios (data not shown). We collected whole plants as voucher specimens for all populations >20 plants but used photographs to voucher smaller populations; specimens are lodged at the Kent State University Herbarium (KE) with accession numbers 64476–64499, 65918–65947 and 66054–66071 (midwestherbaria.org) and photos are available by request from the authors.

### 4.3. Tissue Sampling and DNA Extraction

We collected and dried bud and/or leaf tissue in silica gel from 2 to 66 plants in 91 of the 92 populations surveyed and generally sampled 10% of the censused population size. Plants were sampled systematically such that we collected tissue from every 10th plant counted in the census. If populations were smaller than 20 plants, we collected tissue from all individuals. Dried buds (when possible) or leaf tissue samples were transferred to −80 °C for at least one week before DNA isolation. We extracted DNA from bud and leaf tissue using a modified CTAB/chloroform protocol of Doyle and Doyle [46] and stored the product at −30 °C. We used a 1:10 dilution of the isolated DNA for all subsequent molecular work.

### 4.4. Plastid Sequencing and Microsatellite Genotyping

We used Sanger sequencing to characterize variation in a single pt intergenic region (*psb*K*–rps*16; Figure S1). Because we could not amplify the entire *psb*K*–rps*16 region in a single PCR reaction, we ran two separate reactions with primer pairs 297/426 and 425/427 amplifying into *rps*16 (Figure S1 and Table S4). When we encountered amplification problems, we replaced primer 425 with 330 and 427 with 331 or 15 until amplification was successful. Details about PCR amplification and sequencing conditions are presented in Supplemental Information.

The hypervariable minisatellite motifs (Figure S1) initially appeared to uniquely identify the *psb*K*–rps*16 haplotypes. However, we soon discovered several independently derived microsatellite motifs that differed at mutations elsewhere in the region. Therefore, we subsequently screened the minisatellite motifs for additional samples and obtained complete *psb*K*–rps*16 sequences under three circumstances: when an additional, novel minisatellite was encountered; when one of the independently derived minisatellites was encountered; and as random checks for the uniqueness of minisatellite motifs that were assumed to be unique. We aligned pt sequences using Sequencher 4.10.1 (Gene Codes, Ann Arbor, MI, USA) [47] and manually verified and recorded SNPs and indels for each individual. To correctly identify haplotypes, each sample was BLASTed against a haplotype library of fully characterized *psb*K*–rps*16 voucher sequences. New sequences were manually checked to confirm variable sites and haplotype before being added to the haplotype library. In total, we haplotyped 2–46 individuals (mean = 6.98) from each of 83 *L. siphilitica* populations (total *n* = 579 individuals; Table S1); 142 of these haplotypes were based on complete *psb*K*–rps*16 sequences and 438 on only the minisatellite motifs (see: Supplemental Information).

We used inferred and complete *psb*K*–rps*16 sequences to construct a pairwise distance matrix that excluded the minisatellite locus. We then inferred maximum parsimony trees using PAUP* 4.0b10 [48]. Details about tree construction and outgroup comparisons are found in Supplemental Information. The tree used here is not the same tree as used in Adhikari et al. [14], which was an unrooted median-joining network based on a subset of sequences from our full dataset that was constructed specifically to test for linkage disequilibrium between the pt and mt genomes.

To characterize patterns of nuclear genetic diversity, we screened 1713 individuals from 90 populations, with an average of 18.67 plants per population. We analyzed data from three polymorphic nuclear microsatellite loci: *Lob6*, *Lob9*, and *Lobtri1* (total of 50 unique alleles; Table S2) [49]. We duplexed *Lob6* and *Lob9* for PCR amplification and mixed it with *Lobtri1* PCR product before genotyping. Specific details about PCR amplification conditions and genotyping are presented in Supplemental Information. We manually confirmed all peaks and bins for these data using Genemapper (Applied Biosystems, Carlsbad, CA, USA). Sanger sequencing was carried out on homozygotes for each locus to confirm the fragment length binning. Missing data rates for *Lob6*, *Lob9,* and *Lobtri1* were 1.4%, 1.1%, and 2.0%, respectively.

### 4.5. Statistical Analyses

#### 4.5.1. Geography of Population Size and Sex Ratio in *L. siphilitica*

All maps were generated using the ‘maps’ package [50] in R [51] to plot populations as data points. We used the ‘plotrix’ package [52] in R [51] to visualize population locations as pie charts showing sex ratio (Figure 1) or haplogroup composition (Figure 2). We estimated the degree of spatial autocorrelation in population size and female frequency based on Moran’s I using the package ‘ape’ [53] in R [51]. Significant spatial structure was examined using post hoc linear and non-linear regression analyses between population size, sex ratio, latitude, and longitude in JMP v. 14 (SAS, Cary, NC, USA) [54]. In order to examine central–peripheral dynamics, we categorized populations as either central or peripheral based on two ecoregions (level II) that overlay the center of the species range: 8.2, Central USA Plains, and 8.3, Southeastern USA Plains [32]. We conservatively considered populations in these two ecoregions to be ‘central’ and those outside it ‘peripheral’ (Figure 1). Note that ecoregion 8.3 has a discontinuous distribution and only populations in the Illinois, Indiana, Iowa, Kentucky, Ohio, and Missouri portion of its distribution were considered central. This provided a relatively objective way to categorize populations as central vs. peripheral for discrete tests of the abundant-center model. We tested for differences in population size and sex ratio between central and peripheral populations using 2-tailed *t*-tests.

#### 4.5.2. Relationships between Sex Ratio and Genetic Diversity

Because we found 120 unique pt haplotypes (see: Results), all pt genetic analyses were carried out using 13 inferred phylogenetic haplogroups based on the topology of the maximum parsimony tree (Figure S2). The composition of the 13 haplogroups in this analysis is identical to Adhikari et al. [14], but the relationships among haplogroups differ because of the increased sampling here and the rooted parsimony analysis (see: Supplemental Information).

We calculated metrics of population-genetic diversity for both pt and nuclear markers. We calculated pt haplogroup diversity (*h*) as

(1) $$h=(n\times (1-\sum p_{i}^{2}))\times {\left(n-1\right)}^{-1},$$

where *p_i_* is the frequency of a haplogroup, and *n* is sample size per population [55]. We calculated heterozygosity (*H*_o_) at nuclear microsatellites across all populations and the total number of alleles per population using GenAlEx [56]. We expected *h, H*_o_, and the total number of alleles to increase with female frequency and tested this using linear regressions. We tested whether female plants were more likely to be heterozygous than hermaphrodites using a χ^2^ test in JMP v. 14 (SAS, Cary, NC, USA) [54].

To facilitate categorical analyses, we grouped populations into four groups based on female frequency: 0%, 0.1–10%, 10.1–50%, and >50% female (as in Adhikari et al. [14]). We ran ANOVAs to examine the a priori expectation that *h*, *H*_o_, and the total number of alleles would be higher in populations with more female plants. We used analyses of molecular variance (AMOVAs; Arlequin 3.5 [57]) to see how well sex-ratio groupings explained population diversity and population differentiation. To calculate population differentiation, we used the number of pairwise differences for pt haplogroup data and the *R*_ST_-like sums of squared differences for microsatellite data [58]. We used 2-tailed *t-*tests to assess whether specific haplogroups were associated with different female frequencies.

#### 4.5.3. Estimating Population Differentiation, Gene Flow, and Genetic Drift across the Species Range

To estimate population differentiation based on the distribution of alleles among populations, we calculated *F*_ST_ for both pt haplogroups using Arlequin 3.5 [57] and nuclear microsatellite genotypes using GenAlEx [56]. We estimated the number of null alleles at each microsatellite locus using FreeNA [59]. To look at the number of migrants per generation, we estimated *N*_e_*m* from *F*_ST_ values, where

(2) $$N_{e}m={\left(2\times F_{\mathrm{ST}}\right)}^{-1}-1/2$$

for pt markers [60] and

(3) $$N_{e}m={\left(4\times F_{\mathrm{ST}}\right)}^{-1}-1/4$$

for nuclear markers. To look for evidence of gene flow, we used Mantel correlations to examine isolation by distance in GenAlEx [56]. We used mean pairwise differences between populations to estimate genetic distance for pt haplogroups and pairwise *F*_ST_ values for nuclear microsatellites. We interpolated missing data. For estimates of isolation by distance, we used the package ‘fossil’ [61] to calculate pairwise geographic distances between populations in R [51].

To examine potential effects of genetic drift, we ran regressions between population size and *h, H*_o_, and the total number of alleles, expecting these metrics to decrease with population size (JMP v. 14; SAS, Cary, NC, USA [54]). In addition, we classified small populations as those with fewer than 100 plants (as in Caruso and Case [31]) and grouped populations as large vs. small. We used 1-tailed *t*-tests to assess whether *h, H*_o_, and the total number of alleles were lower in small populations (JMP v. 14; SAS, Cary, NC, USA [54]). We also used AMOVAs to see how well categorical population size explained population diversity and among-population differentiation (Arlequin 3.5 [57]).

To examine the geographic structure of genetic metrics, we used Moran’s I to test for spatial autocorrelation of *h, H_o_,* total number of alleles, and individual pt haplogroups (both presence and relative abundance). Significant Moran’s I tests were followed by linear and non-linear regressions against latitude and longitude on quantitative metrics and with 2-tailed *t*-tests on presence of particular haplogroups. We also used 1-tailed *t*-tests to test the a priori expectation that *h, H*_o_, and the total number of alleles would be higher in central vs. peripheral populations. We used AMOVAs to examine how well central–peripheral groupings explained genetic differences among populations (Arlequin 3.5 [57]).

## Figures and Tables

**Table 1:** Analyses of molecular variance based on *Lobelia siphilitica* plastid sequence data and three nuclear microsatellites. Population differentiation was approximated by pairwise differences for 83 populations and by the *R*_ST_-like sum of squared differences for 90 populations for plastid and nuclear data, respectively. Values represent the percent of variation explained by each grouping. * denotes significance at *p* < 0.05, and *** denotes significance at *p* < 0.001.

AMOVA Group	# of Groups	Among Groups	Among Populations within Groups	Within Populations
Plastid haplogroup (*n* = 83)				
Female frequency	4	4.2	69.8 ***	26.0 ***
Population size	2	0	74.4 ***	26.4 ***
Central vs. peripheral	2	10.2 *	64.8 ***	24.9 ***
Nuclear microsatellites (*n* = 90)				
Female frequency	4	1.0 *	14.4 ***	84.5 ***
Population size	2	0	15.3 ***	84.7 ***
Central vs. peripheral	2	2.1 *	14.0 ***	83.9 ***

**Table 2:** Summary of observed associations between spatial, demographic, and genetic variables among populations of *Lobelia siphilitica*.

	Evidence of Spatial Structure	Variation with Population Size	Variation with Female Frequency
Female frequency	Very strong; 7x higher in south–central populations	None	–
Population size	Slightly smaller at lower latitudes	–	None
Plastid haplo-group diversity	Individual haplogroups structured longitudinally	None	None
Nuclear microsatellite diversity	Weak to none	Slightly more alleles in larger populations	Slightly fewer alleles in populations with no female plants

## Data Availability

Plastid haplogroup and nuclear microsatellite data for individual plants are available here: https://doi.org/10.5061/dryad.vmcvdncvp.

## Supplementary Materials

The following are available online at https://www.mdpi.com/article/10.3390/plants11060825/s1, Supplemental Information: Supporting information about *Lobelia siphilitica* occurrence data, plastid PCR and sequencing methods, nuclear microsatellite PCR and genotyping methods, plastid sequence data details, and nuclear microsatellite data details; Table S1: Demographic and plastid haplogroup information for 92 populations of *Lobelia siphilitica*; Table S2: List of alleles found in 90 populations of *Lobelia siphilitica* at nuclear microsatellite loci *Lob6*, *Lob9*, and *Lobtri1*; Table S3: Descriptive statistics of genetic variability using nuclear microsatellite data for 90 populations of *Lobelia siphilitica*; Table S4: List of primers used for PCR amplification; Figure S1: Diagram of the *psb*K*–rps*16 region of the plastid genome in *Lobelia siphilitica*; Figure S2: Phylogenetic analysis of all complete haplotypes; Figure S3: Correlation between geographic and genetic distances in *Lobelia siphilitica*.
