# Supplementary material for: Do Genetic Drift and Gene Flow Affect the Geographic Distribution of Female Plants in Gynodioecious Lobelia siphilitica?
Source: Plants (Basel). 2022 Mar 20;11(6):825. doi: 10.3390/plants11060825 (PMC8950786; doi:10.3390/plants11060825)
Supplement: Supplementary file 1 [file plants-11-00825-s001.zip › Figure S1.pdf]

Appiah-Madson et al.—*Plants* 2022—Figure S1

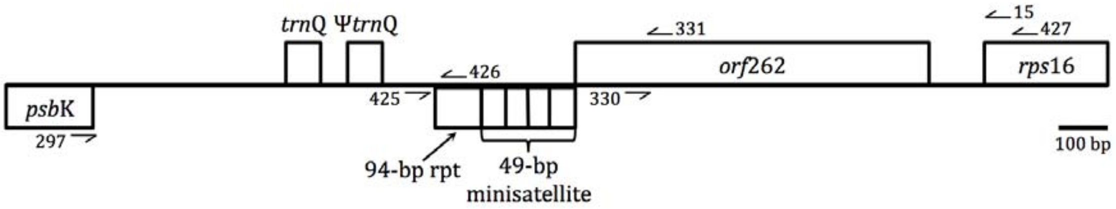

Figure S1. Diagram of the *psb*K–*rps*16 region of the plastid genome in *Lobelia siphilitica,* highlighting the relative positions and orientations of all genes (*psb*K, *rps*16, and two distinct copies of *trn*Q), a 94-bp repeat, an imperfect minisatellite, and unknown open reading frame (*orf*262). Primer sequences are listed in Table S4. Drawing is to scale.
