# Supplementary material for: Do Genetic Drift and Gene Flow Affect the Geographic Distribution of Female Plants in Gynodioecious Lobelia siphilitica?
Source: Plants (Basel). 2022 Mar 20;11(6):825. doi: 10.3390/plants11060825 (PMC8950786; doi:10.3390/plants11060825)
Supplement: Supplementary file 1 [file plants-11-00825-s001.zip › Figure S2.pdf]

Appiah-Madson et al.—*Plants* 2022—Figure S2

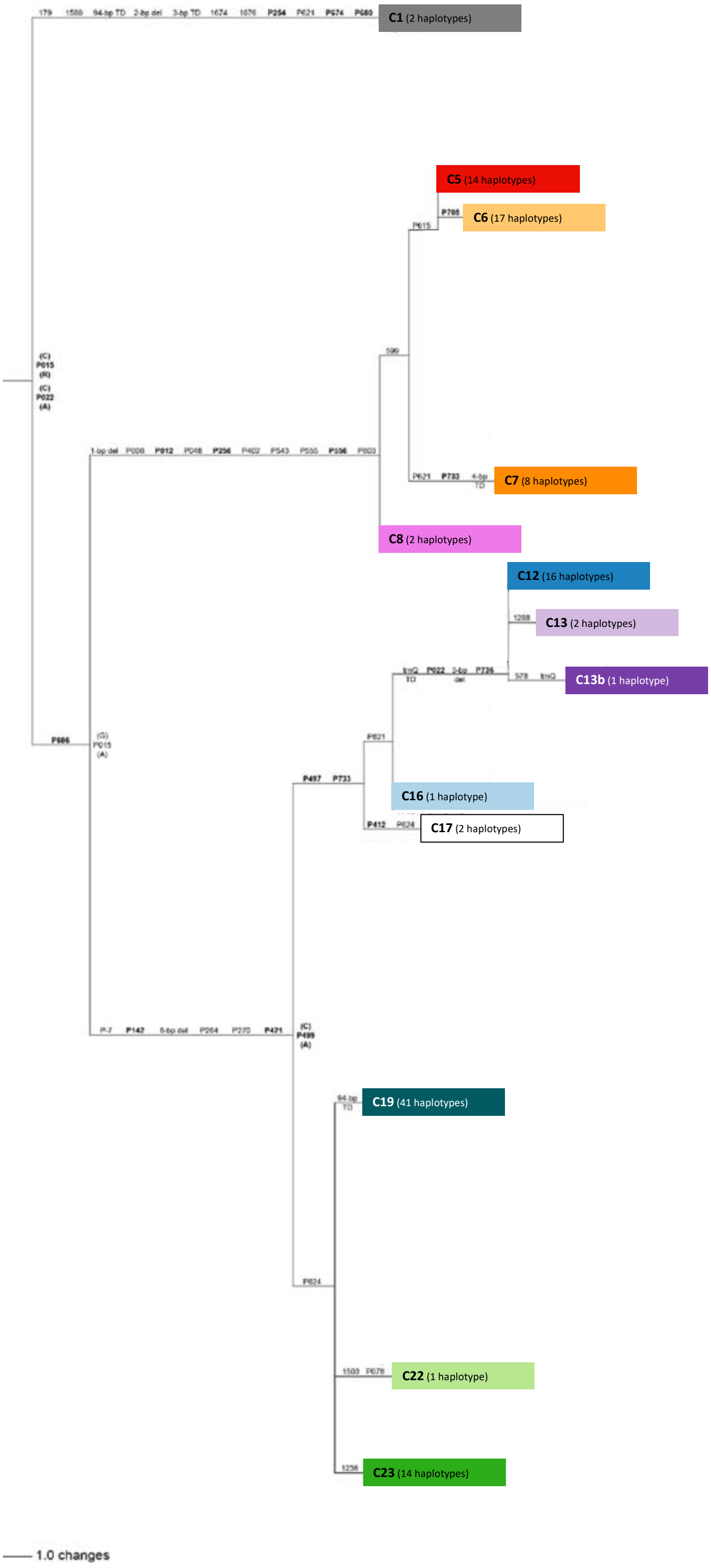

Figure S2. Phylogenetic analysis of all complete haplotypes. Characters represented by numbers indicate the position of point mutations in the multiple sequence alignment. Numbers preceded by a ‘P’ indicate the position of point mutations in *orf*262. Negative numbers preceded by a ‘P’ indicate the position of point mutations in positions upstream of *orf*262. Point mutations that cannot be unambiguously polarized with the current data are placed at the respective nodes, with the alternative nucleotide character states in the branches above and below those nodes shown in parentheses. (bp = base-pair; CE = concerted evolution; del = deletion; TD = tandem duplication.)
