# Supplementary material for: Do Genetic Drift and Gene Flow Affect the Geographic Distribution of Female Plants in Gynodioecious Lobelia siphilitica?
Source: Plants (Basel). 2022 Mar 20;11(6):825. doi: 10.3390/plants11060825 (PMC8950786; doi:10.3390/plants11060825)
Supplement: Supplementary file 1 [file plants-11-00825-s001.zip › Figure S3.pdf]

Appiah-Madson et al.—*Plants* 2022—Figure S3

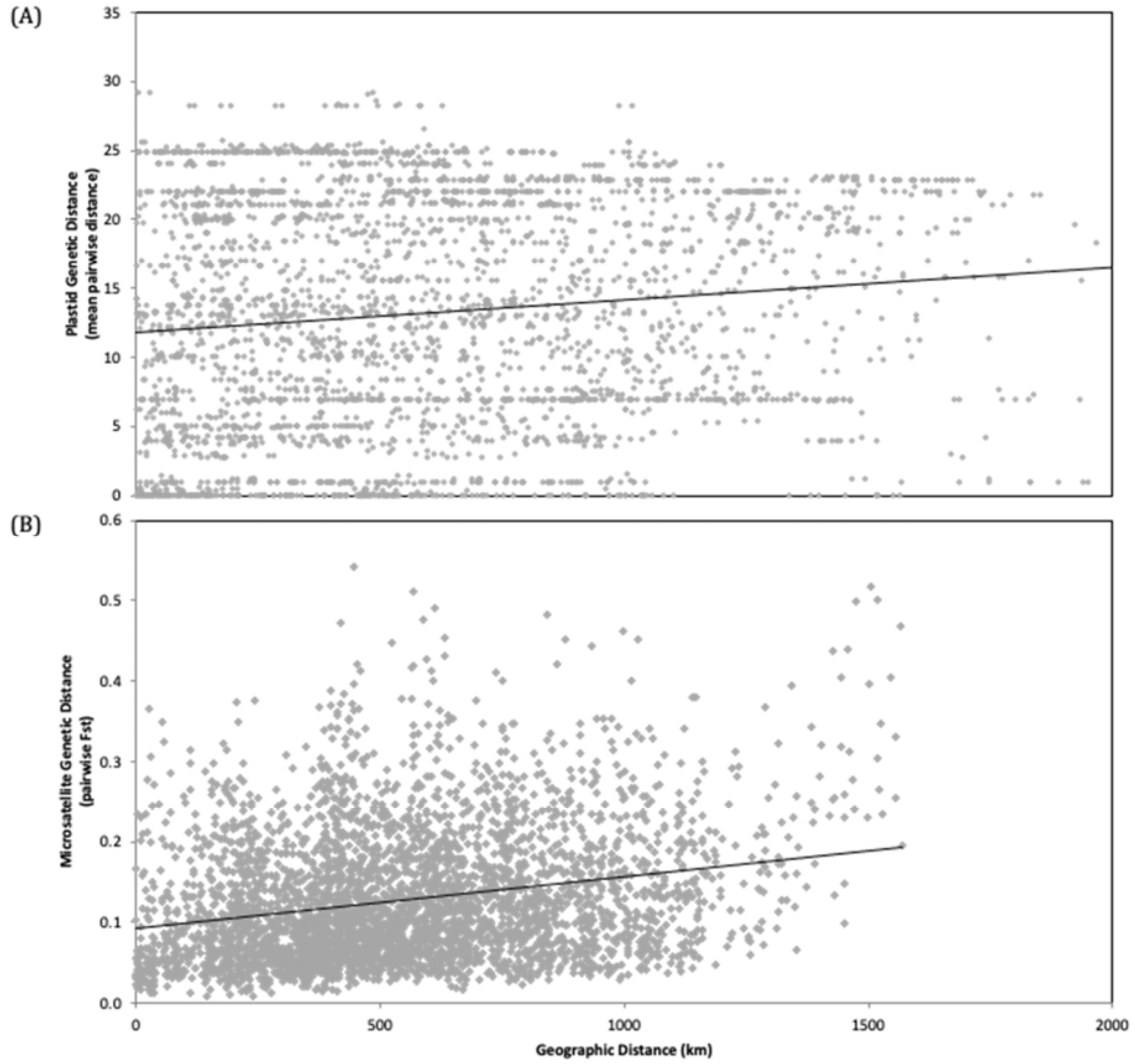

Figure S3. Correlation between geographic and genetic distances in *Lobelia siphilitica* based on (A) mean pairwise differences using 13 plastid haplogroups (Mantel correlation, $r = 0.112$, df = 81, $P = 0.01$) and (B) pairwise F_{ST} using nuclear microsatellite data (Mantel correlation, $r = 0.246$, df = 88, $P = 0.01$).
