# Supplementary material for: Do Genetic Drift and Gene Flow Affect the Geographic Distribution of Female Plants in Gynodioecious Lobelia siphilitica?
Source: Plants (Basel). 2022 Mar 20;11(6):825. doi: 10.3390/plants11060825 (PMC8950786; doi:10.3390/plants11060825)
Supplement: Supplementary file 1 [file plants-11-00825-s001.zip › Supplemental Information_Final.pdf]

Supplemental Information. Supporting information about *Lobelia siphilitica* occurrence data, plastid PCR and sequencing methods, nuclear microsatellite PCR and genotyping methods, plastid sequence data details, and nuclear microsatellite data details.

***Lobelia siphilitica* occurrence data—**Shaded US counties in Figure 1 show the distribution of *L. siphilitica* according to the USDA PLANTS Database (http://plants.usda.gov), supplemented with records from 25 herbaria (BHSC, BUT, CLM, EKY, GRI, IND, ISC, ISM, JFBM, JMUH, KE, MISS, MO, MU, NYBG, ODU, SIU, TENN, UOS, US, USCH, VPI, VT, WILLI, WVIT) and four flora guides. These records were also used to locate populations in the field in both sampling years.

***Plastid PCR and sequencing methods*—**We used the following master mix for PCR amplification: 15.4 µL aH_2O, 2.5 µL 10x Rxn Buffer, 2.0 µL $MgCl_2$, 0.5 µL 10 mM dNTPs, 1.0 µL 5 µM of each primer, 0.1 µL taq, and 2.5 µL DNA template. Amplification was carried out according to the following profile: 3 min at 90°C, 35 cycles of 94°C for 30 s, 53°C for 20 s, and 72°C for 1 min 30 s, with a final extension for 5 min at 72°C. MacrogenUSA Corp. (Rockville, Maryland, USA) carried out purification and sequencing using an ABI 3730XL (Applied Biosystems, Carlsbad, California, USA). We provided primers 425, 427, 297, 426, and 331 at 5 µM for sequencing reactions (see: Table S4 for primer details).

***Nuclear microsatellite PCR and genotyping methods*—**We duplexed *Lob6* and *Lob9* using the following master mix: 1.94 µL aH_2O, 1.38 µL 5x Rxn Buffer, 0.52 µL $MgCl_2$, 0.65 µL 2.5 µM dNTPs, 0.13 µL 10 µM of each primer, 0.02 µL BSA, 0.065 µL taq, and 2 µL DNA template. For *Lobtri1* we used 1.96 µL aH_2O, 1.2 µL 5x Rxn Buffer, 0.48 µL $MgCl_2$, 1.2 µL 2.5 µM dNTPs, 0.12 µL 10 µM *Lobtri1* primers, 0.02 µL BSA, 0.06 µL taq, and 1.8 µL DNA template. We carried out amplification according to the following profile: 4 min at 95°C, 40 cycles (*Lob 6/9*) or 44 cycles (*Lobtri1*) of 95°C for 30 s, 51°C (*Lob*

6/9) or 63°C (*Lobtri1*) for 30 s, and 72°C for 30 s, with a final extension for 7 min at 72°C. We mixed 1.7 µL of *Lob6/9* PCR product with 2 µL *Lobtri1* PCR product and diluted it with 6.3 µL aH_2O before sending it to the DNA Core Sequencing Facility (Urbana, Illinois, USA) for genotyping on an ABI 3730XL (Applied Biosystems, Carlsbad, California, USA).

To call and manually confirm peaks and bin alleles for the nuclear microsatellite data, we used Genemapper (Applied Biosystems, Carlsbad, California, USA). As *Lob6* and *Lob9* are both tetramers, samples were allowed a 1.83 bp margin of error, but trimer *Lobtri1* was only allowed a 1.3 bp margin of error. Samples that did not fall within bins were reamplified using the same DNA template and rerun. Sanger sequencing was carried out on homozygotes for each locus (as described above) to confirm the fragment length binning. We adjusted the threshold for calling peaks such that samples were only considered heterozygous at a particular locus if the two peaks were within 50% of each other's heights. We analyzed the data both with and without this adjustment with no effect on the inferred patterns. Missing data rates for *Lob6*, *Lob9*, and *Lobtri1* were 1.4%, 1.1%, and 2.0%, respectively.

***Plastid sequence data details*—**The population and voucher information were recorded for the tissue samples used to generate the complete *psb*K–*rps*16 sequences and these representative full sequences were named based on the hypervariable minisatellite motifs. Initial complete *psb*K–*rps*16 sequencing results indicated that these minisatellite motifs varied in complexity and not all motifs were uniquely derived (based on diagnostic mutations elsewhere in the *psb*K–*rps*16 region). Numeric suffixes were added to distinguish among motifs inferred to have evolved independently and alphabetic suffixes were added to distinguish among *psb*K–*rps*16 sequences with a shared motif but are distinguished by mutations elsewhere in the region. Because of the ongoing trafficking of 'foreign' (= extra-plastid) DNA bearing large open reading frames (ORFs; putative protein-coding genes) in the *psb*K–*rps*16 region, the outgroup species (*L. cardinalis*, GenBank accession MF770634 and *L. puberula*, GenBank accession MF770611) only share the canonical plastid (pt) genes and the 3' portion of *orf*262, which initially limited our ability

to polarize several mutations in an unrooted haplotype network that were critical for establishing the basal relationships among the *L. siphilitica psb*K–*rps*16 sequences in a directed maximum parsimony tree obtained using PAUP* 4.0b10. Of specific concern was the C1 haplogroup (with its numerous distinctive mutations) that was recovered from the BT population in Minnesota because *L. siphilitica* var. *ludoviciana* is known to occur in Minnesota. In the context of multiple North American outgroup species that were previously or more recently sequenced, maximum parsimony analysis of complete plastomes from a Colorado sample of *L. siphilitica* var. *ludoviciana* (Clark 3806; C1 haplogroup), a sample of the cultivated hybrid *L.* × *speciosa* (Lammers 8708; C5 haplotype; with maternal parent *L. siphilitica* var. *siphilitica* and paternal parent *L. cardinalis*), and two Indiana samples of *L. siphilitica* var. *siphilitica* (Knox 5089A and Knox 5089C; C5 and C12 haplogroups, respectively) established that var. *ludoviciana* is sister to var. *siphilitica* (E.B. Knox, unpublished data). This contextual information allowed the point mutations, deletions (del), tandem duplications (TD), and the inferred concerted evolution (CE) and sequence conversion events in the *psb*K–*rps*16 region to be manually mapped onto a tree that does not yet include the variation within the hypervariable minisatellite that is responsible for the variation among the motifs (Figure S2). The resulting tree length, including both of the known C1 haplotypes is 79 steps, the CI is 0.9483, and the RI is 0.9973. Four point mutations in *orf*262 are mapped at a node instead of one of the branches because they cannot be polarized, and the alternative nucleotides are presented in parentheses above and below the name of the point mutation.

Out of 142 complete sequences of the pt *psb*K–*rps*16 region, we found 120 unique haplotypes. Total length varied between 2029–2555 bp, with 36 variable sites, 12 indels (1–30 bp in length), variable copy numbers of *trn*Q, Ψ*trn*Q, and a 94-bp repeat, and an imperfect minisatellite locus (Figure S1). The majority (75%) of the variable sites, and seven of the 12 indels, were in the reading frame of *orf*262. The minisatellite locus, located immediately upstream from *orf*262, was imperfect and hypervariable. Unique haplotypes contained 1–13 minisatellite repeat units, each 48–54 bp long. We found 22 unique repeat unit sequences, and 98 unique combinations of these units.

Phylogenetic analysis excluding the variation in the minisatellite yielded a single phylogenetic tree containing 23 unique clades, each containing 1–29 unique haplotypes. The 23 terminal clades were condensed into 13 haplogroups by collapsing nested clades defined by homoplasious variation in several of the tandem repeats. The 13 inferred haplogroups used for analysis were supported by 35 SNPs, three indels, and three different versions of the 94-bp repeat (Figure S2).

Nuclear microsatellite data details**—**We detected 12, 17, and 21 alleles for *Lob6*, *Lob9*, and *Lobtri1*, respectively, for a total of 50 unique alleles. Allele sizes were contiguous for *Lob6* and *Lob9*, but not for *Lobtri1* (Table S2). The average number of alleles per population (N_a) was 4.200, 4.644, and 5.811 while the average effective number of alleles (N_e) was 2.600, 2.766, and 3.513 for *Lob6*, *Lob9*, and *Lobtri1*, respectively (Table S3). We found evidence of null alleles, but they did not significantly affect estimates of population differentiation (F_{ST}; data not shown). In 25 populations, one or more loci were out of Hardy-Weinberg equilibrium, and 32 populations had loci in apparent linkage disequilibrium, but this was not related to population size or sex ratio (data not shown). All but three populations were polymorphic at all three loci. Observed (H_o) and expected (H_e) heterozygosities were moderate (Table S3): H_o was 0.570 averaged across loci (range among populations = 0.200–0.917), which was similar to H_e ($\bar{x}$ = 0.606; range = 0.247–0.764). Across populations, pt haplogroup diversity (*h*) was significantly correlated with nuclear allelic diversity (*h* vs. N_a: linear regression, $r = 0.289$, df = 80, $P = 0.0085$; *h* vs. N_e: linear regression, $r = 0.225$, df = 80, $P = 0.042$) but not with heterozygosity (*h* vs. H_o: linear regression, $r = 0.04$, df = 80, $P = 0.10$).
