# Supplementary material for: Do Genetic Drift and Gene Flow Affect the Geographic Distribution of Female Plants in Gynodioecious Lobelia siphilitica?
Source: Plants (Basel). 2022 Mar 20;11(6):825. doi: 10.3390/plants11060825 (PMC8950786; doi:10.3390/plants11060825)
Supplement: Supplementary file 1 [file plants-11-00825-s001.zip › Table S2.pdf]

Table S2. List of alleles found in 90 populations of *Lobelia siphilitica* at nuclear microsatellite loci *Lob6*, *Lob9*, and *Lobtri1*.

Lob6 (GTAT)$_{2\text{-}13}$	*Lob9* (CATA)$_{1\text{-}17}$	*Lobtri1* (CAA)$_{2\text{-}24}$
5	214	310
99	218	313
103	222	316
107	226	319
111	230	322
115	234	325
119	238	328
123	242	331
127	246	334
131	250	337
135	254	340
139	258	343
	262	346
	266	349
	270	352
	274	355
	278	358
		361
		364

370
376
