# Supplementary material for: Do Genetic Drift and Gene Flow Affect the Geographic Distribution of Female Plants in Gynodioecious Lobelia siphilitica?
Source: Plants (Basel). 2022 Mar 20;11(6):825. doi: 10.3390/plants11060825 (PMC8950786; doi:10.3390/plants11060825)
Supplement: Supplementary file 1 [file plants-11-00825-s001.zip › Table S3.pdf]

Table S3. Descriptive statistics of genetic variability using nuclear microsatellite data for 90 populations of *Lobelia siphilitica*. Average and standard error of the number of samples run for each population (N), the number of alleles (N_a), the number of effective alleles (N_e), Shannon's information index (I), observed heterozygosity (H_o), expected heterozygosity (H_e), unbiased expected heterozygosity (UH_e), and the fixation index (F), as well as F-statistics (F_{IS}, F_{IT}, F_{ST}) and the estimated migration rate ($N_e m$) based on three nuclear microsatellite loci as calculated using GenAlEx. Missing data rates for *Lob6*, *Lob9,* and *Lobtri1* were 1.4%, 1.1%, and 2.0%, respectively.

Loci	N	N_a	N_e	I	H_o	H_e	UH_e	F	F_{IS}	F_{IT}	F_{ST}	$N_e m$
Lob6	18.689	4.200	2.600	1.048	0.553	0.562	0.588	0.004	0.015	0.212	0.199	1.008
Lob9	18.756	4.644	2.766	1.120	0.552	0.582	0.605	0.042	0.051	0.274	0.235	0.813
Lobtri1	18.567	5.811	3.513	1.354	0.604	0.665	0.695	0.067	0.091	0.301	0.231	0.832
Mean	18.670	4.885	2.960	1.174	0.570	0.603	0.629	0.037	0.053	0.262	0.222	0.884
SE	0.716	0.123	0.070	0.025	0.013	0.010	0.011	0.018	0.022	0.027	0.011	0.062
