# Supplementary material for: Do Genetic Drift and Gene Flow Affect the Geographic Distribution of Female Plants in Gynodioecious Lobelia siphilitica?
Source: Plants (Basel). 2022 Mar 20;11(6):825. doi: 10.3390/plants11060825 (PMC8950786; doi:10.3390/plants11060825)
Supplement: Supplementary file 1 [file plants-11-00825-s001.zip › Table S4.pdf]

Table S4. List of primers used for PCR amplification for both plastid sequencing and nuclear microsatellite genotyping in *Lobelia siphilitica*.

Number	Primer name	Sequence (5' to 3')	Aligned fragment length (bp)
Plastid sequencing			
426	LsA012641F(426)B2	CAAATAAGAGTATATGCACGAATAGC	426 + 297: 872–1014
297	Lt013173R(297)*psb*K	AAAAAAGCATAGGCCTCGGG	
427	Lt011571F(427)*rps*16	GCAACGATTYGATAAGCCGC	427 + 425: 1299–1825
425	LsA012737R(425)B2	GGGTTTTTGAAGTTCCATCGG	
330	LsA012115R(330)B2	GGTACGGGCTATTGCCGCTGG	427 + 330: 643–682
331	LsA011964F(331)B2	CCGTTCCGGTGTTGCCCTACC	425 + 331: 573–1099
15	Interseqfor10(15)	AGTCATTGGTTCAGTCGGTA	425 + 15: 1178–1704
Microsatellite genotyping			
*Lob6*F	*Lob6*40L	TTTGCCATCAGGATATTCAAAG	95–139
*Lob6*R	*Lob6*40RHEX	CAACATGATGCTCATGGGACT	
*Lob9*F	*Lob9*50L	GGCAATTTGGTAATCCCTCTT	214–278
*Lob9*R	*Lob9*50R6FAM	CGACATCCCATTGTCAACCT	
*Lobtri1*F	*Lobtri1*F	CCCCAAGTGATTGGGACTAA	310–376

*Lobtri1*R	*Lobtri1*151RHEX	CGACATAACGGAAAAGGAACA
